# A review of frustrated Lewis pair enabled monoselective C–F bond activation

**DOI:** 10.1039/d3sc06485a

**Published:** 2024-01-16

**Authors:** Kenneth Lye, Rowan D. Young

**Affiliations:** a Department of Chemistry, National University of Singapore 117543 Singapore; b School of Chemistry and Molecular Biosciences, The University of Queensland St Lucia 4072 Australia rowan.young@uq.edu.au

## Abstract

Frustrated Lewis pair (FLP) bond activation chemistry has greatly developed over the last two decades since the seminal report of metal-free reversible hydrogen activation. Recently, FLP systems have been utilized to allow monoselective C–F bond activation (at equivalent sites) in polyfluoroalkanes. The problem of ‘over-defluorination’ in the functionalization of polyfluoroalkanes (where multiple fluoro-positions are uncontrollably functionalized) has been a long-standing chemical problem in fluorocarbon chemistry for over 80 years. FLP mediated monoselective C–F bond activation is complementary to other solutions developed to address ‘over-defluorination’ and offers several advantages and unique opportunities. This perspective highlights some of these advantages and opportunities and places the development of FLP mediated C–F bond activation into the context of the wider effort to overcome ‘over-defluorination’.

## Abstract 2

Functionalisation of polyfluorocarbon positions generally suffers from over-defluorination. Frustrated Lewis pairs (FLPs) offer a unique solution to this problem allowing direct access to a wide range of 2nd generation fluorocarbon products.

## Graphical abstract

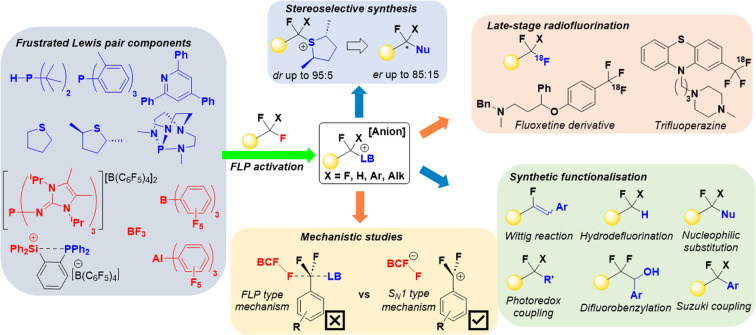

## Introduction

**Fig. 1:**
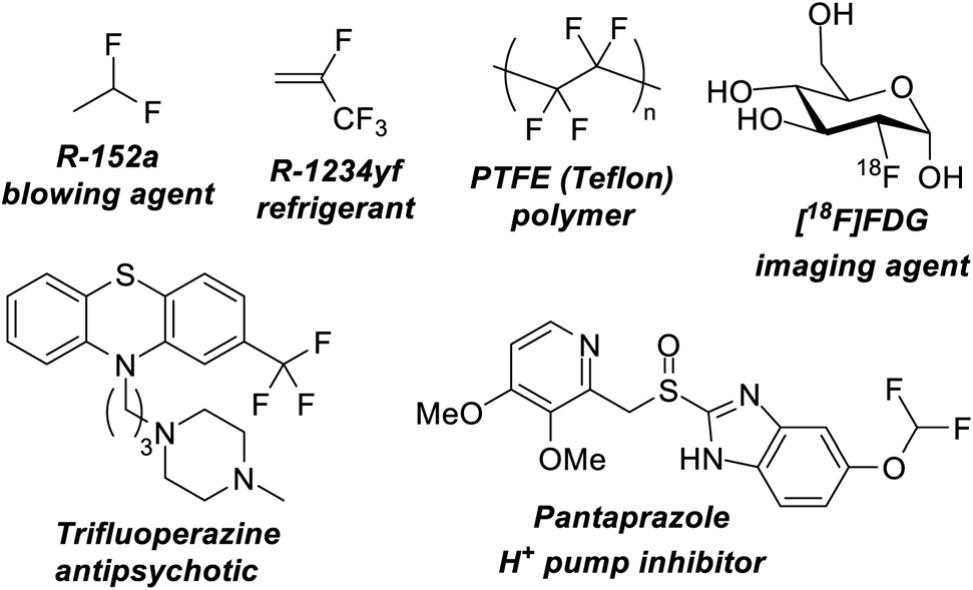
Fluorocarbons containing sp^3^ fluorine positions are vitally important to many modern technologies and can act as blowing agents, refrigerants, polymers, imaging agents and pharmaceuticals.

Over the last century organofluorine chemistry has become intrinsically important in an array of fields.^1^ Fluorocarbons are invaluable as refrigerants and blowing agents, in polymer and materials chemistry, in pharmaceuticals and agrochemicals, in imaging science and radiology, and in lubricants and surfactants (Fig. 1). Despite examples of environmental and health concerns for certain fluorocarbons, the fluorocarbon market continues to expand.^2^ For example, 4th generation refrigerants and blowing agents based on hydrofluoroolefins are being introduced to replace high global warming potential hydrofluorocarbons, and fluorocarbons represent over 20% of marketed pharmaceuticals and 50% of marketed agrochemicals.^3^ The growth of the fluorocarbon sector is contingent upon the unique properties that fluorine containing motifs possess. The high bond dissociation energies of C–F bonds renders them stable to denigratory chemical and biological processes, the highly polarized C–F bond promotes solubility, ‘fluorine’ effects give rise to unique preferred geometries, and fluorine containing motifs are excellent bioisosteres for hydroxyl, keto, methyl and amido groups (*inter alia*).^4^

Consequently, methods to incorporate fluorine into sp^3^ C–F positions are highly developed.^5^ Early methods relied upon using hydrogen fluoride (HF) or HF surrogates, however, a number of methods have been developed that utilize sulfonyl fluorides, electrophilic fluorine and fluoroalkylation. Such methods constitute a ‘bottom-up’ approach to the synthesis of fluorocarbons.

**Fig. 2:**
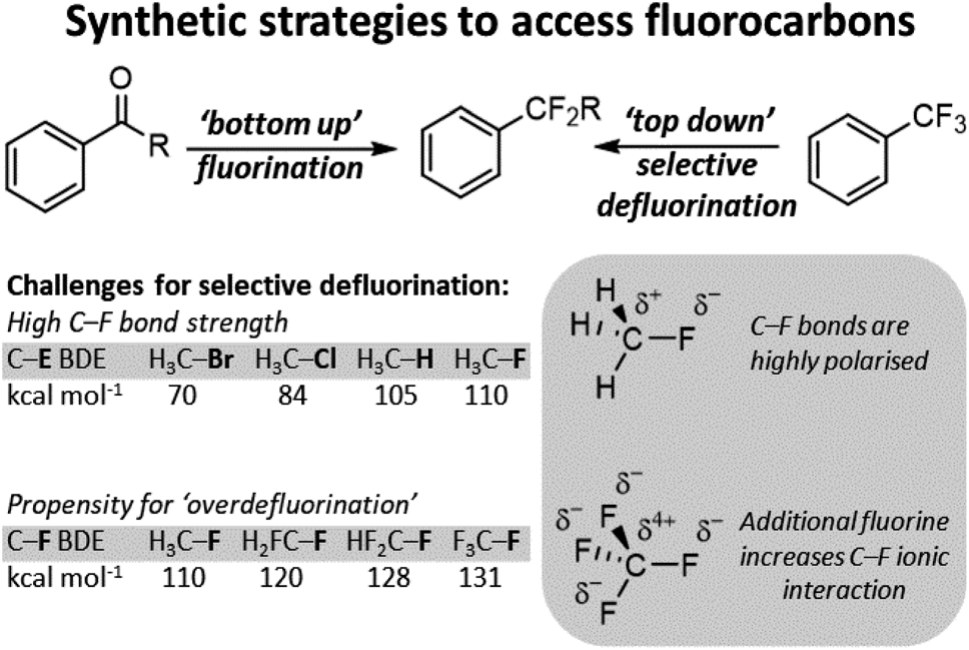
Fluorocarbons can be accessed *via* a ‘bottom-up’ approach where fluorine is added to substrate or *via* a ‘top-down’ approach where fluorine is selectively removed from a polyfluorocarbon to form a second-generation fluorocarbon. Top-down approaches must overcome the high C–F bond strength and the propensity for polyfluorocarbon positions to ‘over-defluorinate’.

With the wide availability of fluorocarbons, methods for C–F functionalization have also been developed.^6^ In modern chemistry, such methods render sp^3^ C–F bonds as versatile synthetic handles to access a wide range of subsequent chemical groups. In general, the majority of sp^3^ C–F functionalization technologies are conceived for carbon positions with a single appended fluoride. Indeed, most of these synthetic strategies cannot be applied to the functionalization of a single fluoride in polyfluorocarbons containing equivalent C–F positions. This is due to the higher stability of more fluorinated carbon positions arising from increased polarity of the C–F bond. This renders functionalized products much more reactive than the parent polyfluorocarbon starting materials and results in ‘overdefluorination’ (Fig. 2).

More recently, attention has turned to overcoming the hurdle of ‘over-defluorination’ giving opportunities for selective activation of single sp^3^ C–F bonds in polyfluorocarbons as a ‘top-down’ route to accessing new fluorocarbons.^7^ This approach has a number of advantages, namely; (i) a vast array of 2nd generation fluorocarbons are readily accessible from a single parent fluorocarbon. In most instances, these 2nd generation fluorocarbons contain F_*n*−1_ as compared to the parent fluorocarbon, (ii) the use of fluorinating reagents are avoided. This avoids employing potentially harmful reagents with many fluorinating reagents generating hydrogen fluoride as a side-product, (iii) it provides the ability for more specific chemo and/or regioselectivity as compared to utilizing fluorinating reagents with the pre-existing polyfluorocarbon group dictating site selection, (iv) it allows for late-stage functionalization and derivatization, and (v) it allows for a wide variety of functional group installation influenced by the method of selective C–F functionalization employed. In most instances each selective C–F functionalization method introduces restrictions on both the fluorocarbon substrates employed and the functionalization that is possible.

**Fig. 3:**
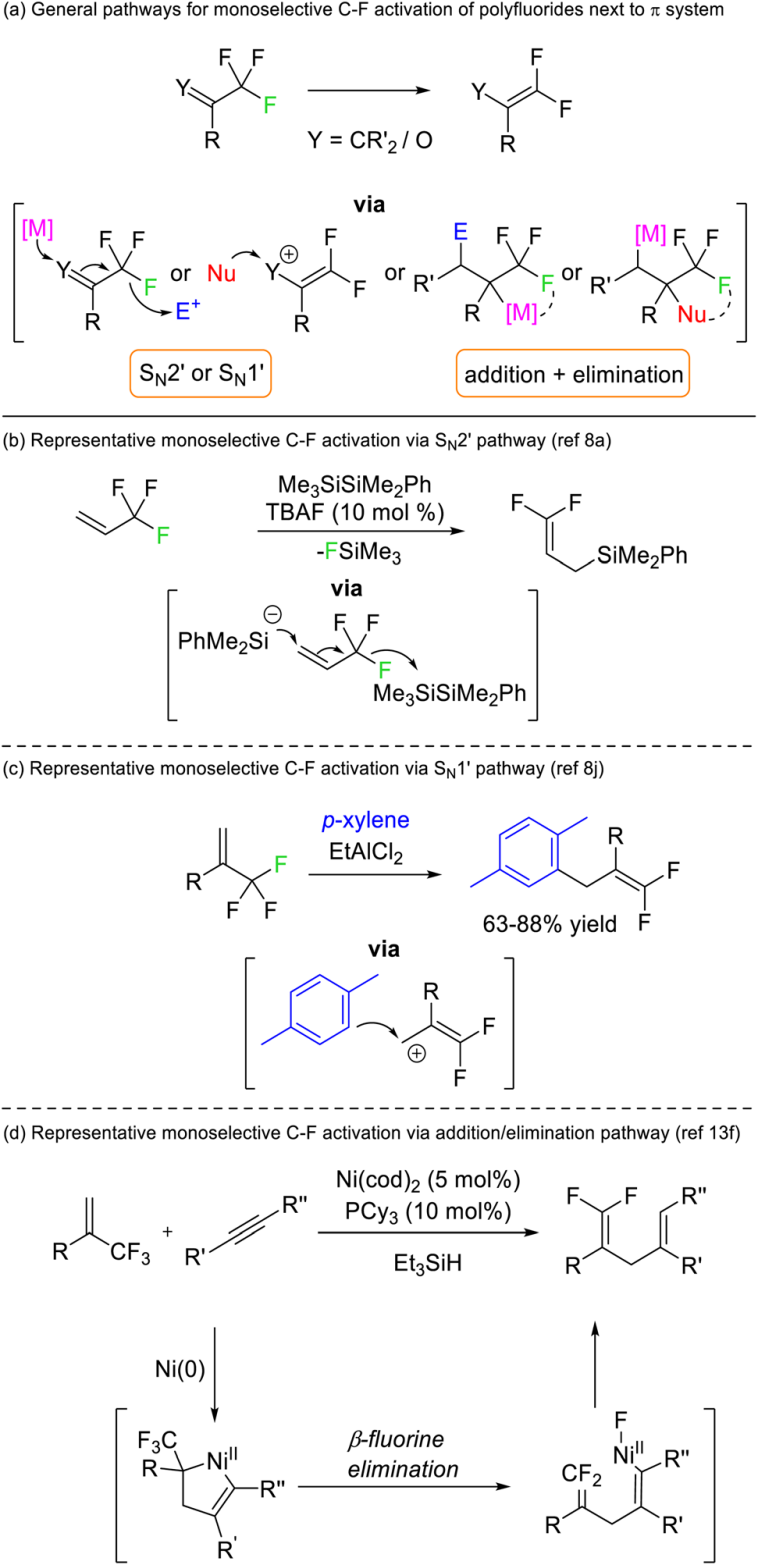
Selective C–F bond activation of CF_3_ and CF_2_ groups adjacent to alkene or carbonyl positions generates fluoroalkene products that possess stable sp^2^ C–F bonds. E = Electrophile, Nu = nucleophile, M = metal, TBA = tetrabutylammonium.

Early success for selective C–F functionalization was achieved by Hiyama through S_N_2′ substitution reactions of trifluoromethyl styrenes with silyl anion nucleophiles (Fig. 3b). The reaction relied upon the transformation of the product C–F bonds to sp^2^ hybridisation rendering them thermodynamically more stable than the sp^3^ C–F bonds in the starting material. Such a strategy has been widely used for 3,3,3-trifluoroallyls and trifluoromethyl ketones but is contingent upon an adjacent π-system (vinyl or carbonyl) (Fig. 3a).^8^ Similar transformations are possible *via* alternative mechanistic pathways (*e.g.* S_N_1′, addition/elimination, see Fig. 3c and d) but rely on the same thermodynamic preference for sp^2^ C–F bonds over sp^3^ C–F bonds.

**Fig. 4:**
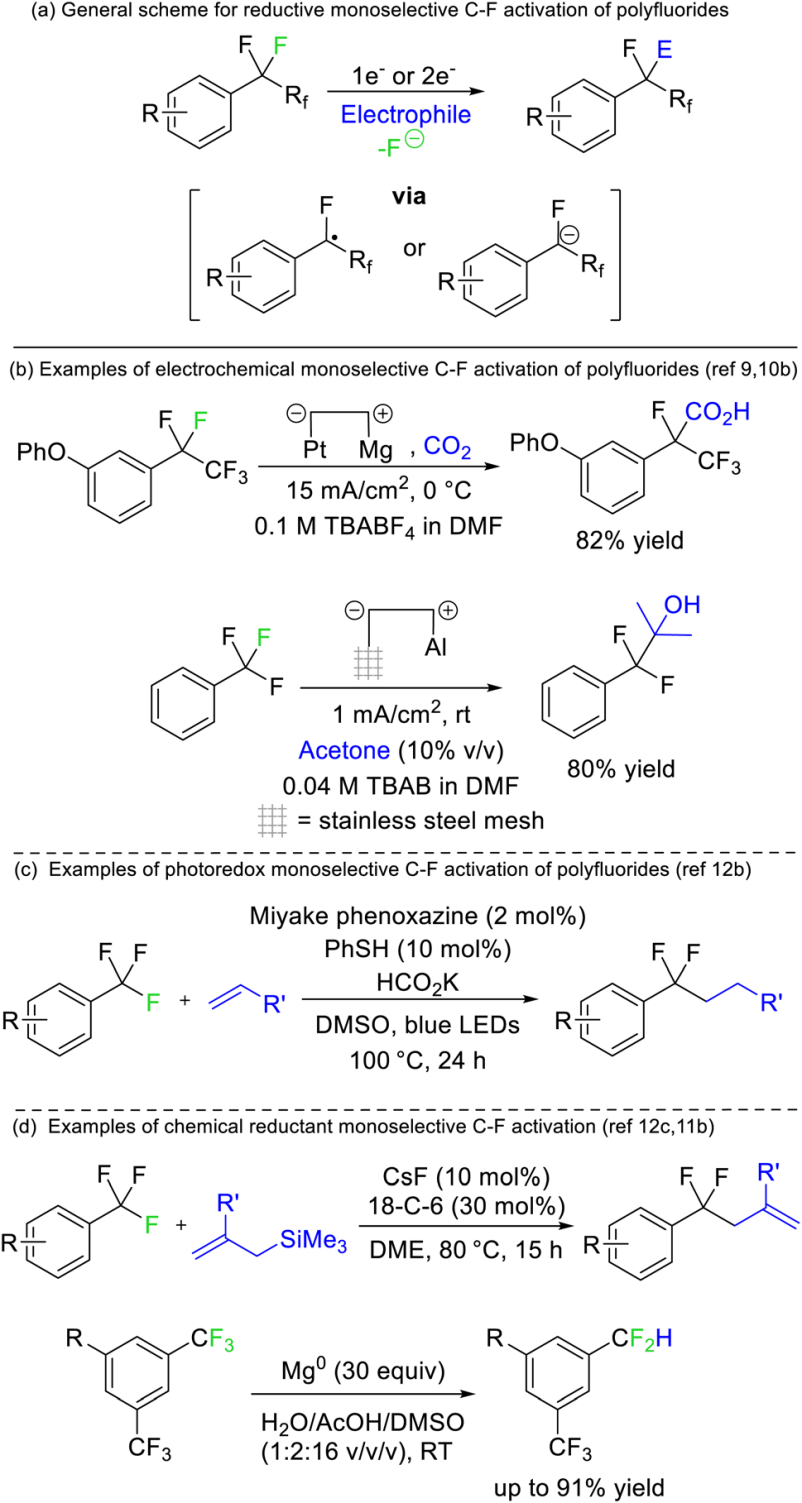
One or two electron reduction of CF_3_ groups supported by arenes, amides and esters allows selective defluorination as the functionalized products of such reactions have higher reduction potentials than the fluorocarbon starting materials. The reduction can be achieved; (b) electrochemically, (c) photolytically or (d) chemically. TBA = Tetrabutylammonium, 18-C-6 = 18-crown-6 ether.

Périchon later demonstrated that benzotrifluorides were more prone to electrochemical reduction than difluoromethylene derivatives owing to the higher electron withdrawing ability of the CF_3_ group (Fig. 4b). Such a strategy allowed the resultant phenyl difluoromethylide to attack acetone, *N*,*N*-dimethylformamide and carbon dioxide electrophiles.^9^ The synthetic utility of this approach has recently been revived by a number of groups.^10^ The same reaction has also been reported using stoichiometric chemical reductants (Fig. 4d)^11^ and a similar approach is possible based on single electron reduction by photoreductive dyes or homolysis of silicon-element bonds (Fig. 4c).^12^ Currently, reductive strategies are most efficient with electron deficient benzotrifluoride and trifluoromethyl esters/amides. Electrophilic coupling has been demonstrated for protons, deuterons, carbon dioxide, amides, alkenes, ketones/aldehydes, and imines. However, radical difluoromethylbenzenes generated from this approach can also be utilized in transition metal catalysed and radical–radical coupling reactions to install aryls, sulfides, oxides, selenides and amines (Fig. 4).

**Fig. 5:**
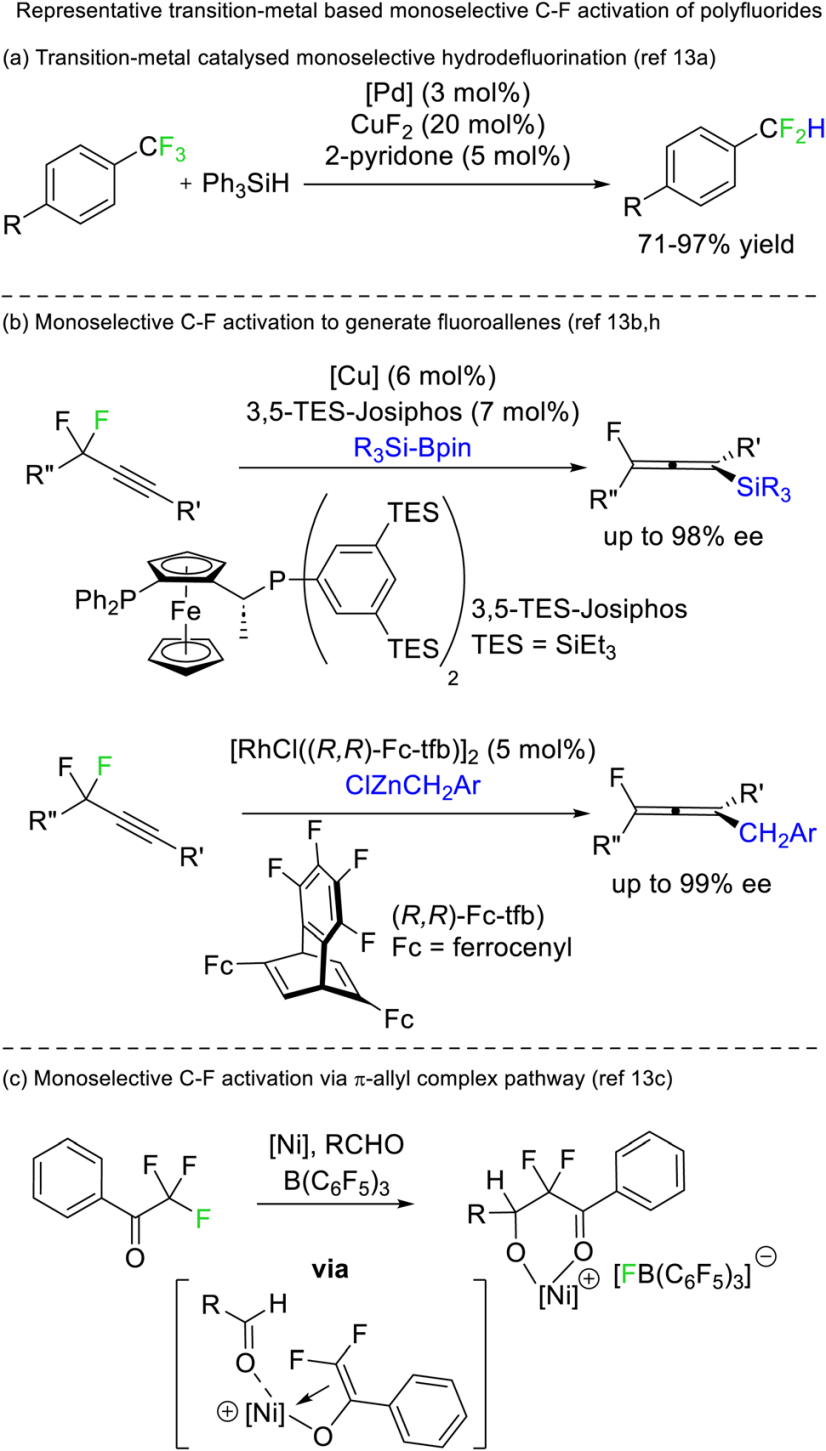
Transition metals can mediate selective defluorination (stoichiometrically and catalytically). Recently, transition metal catalysis has allowed for the enantioselective generation of chiral fluorides from achiral difluorides.

A limited number of reports exist for selective defluorination of difluoromethyl and trifluoromethyl groups using metal catalysis (Fig. 5).^13^ Importantly, these reports include the first instances of access to stereoenriched fluorocarbons from achiral fluorocarbon starting materials (Fig. 5b).

**Fig. 6:**
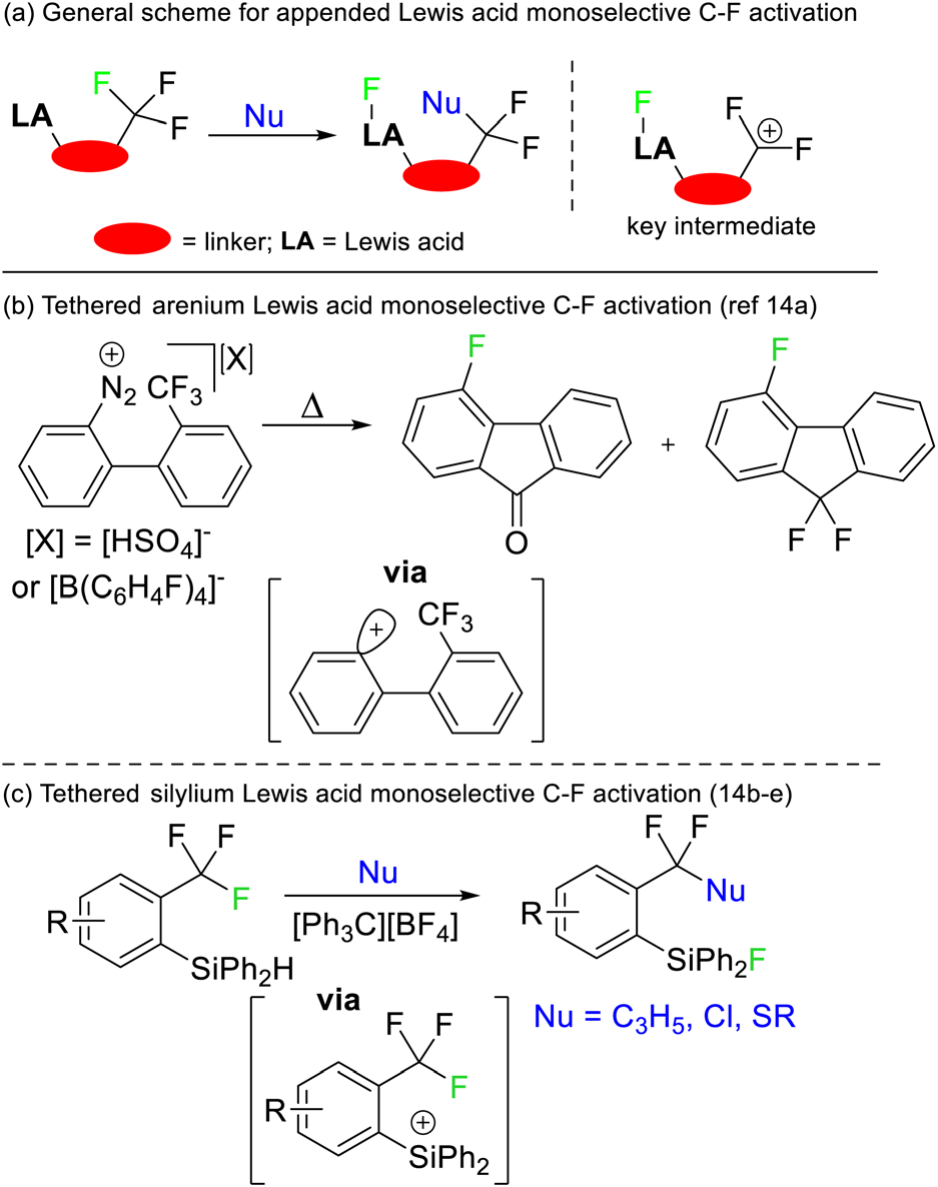
Strong Lewis acids tethered in close proximity to CF_3_ groups allow for kinetically controlled selective defluorination. This approach was first reported by Lectka and has been subsequently developed by Yoshida and Hosoya. Nu = nucleophile.

Selective defluorination of benzotrifluorides has also been mediated by strong Lewis acids. Kinetic selection strategies have been based on tethered Lewis acid sites encroaching the trifluoromethyl group (Fig. 6a). As such, the Lewis acid attacks the most spatially accessible C–F bond rather than the weakest C–F bond. Such an approach was first demonstrated by Lectka in 1997 utilising arenium Lewis acids generated from diazonium precursors (Fig. 6b) but has been refined to be synthetically useful more recently by Yoshida and Hosoya (Fig. 6c).^14^

Generally, the methods for selective defluorination introduced above suffer from limited substrate scope and/or functionalization possibilities. These strategies cannot effect selective defluorination in difluoromethyl or trifluoromethyl alkanes (*e.g.* 1,1-difluoroethane), in difluoromethyl or trifluoromethyl groups attached to heteroatoms (*e.g.* difluorothiomethoxybenzene or trifluoromethoxybenzene) or between chemically equivalent C–F bonds at distal positions (*e.g.* 1,3-difluoropropane). In contrast, the application of frustrated Lewis pairs to the problem of selective defluorination has demonstrated a very wide substrate scope of polyfluorocarbons and allows a vast array of functionalization opportunities, including applications in stereoselective defluorination and radiosynthesis.

## Frustrated Lewis pairs

**Fig. 7:**
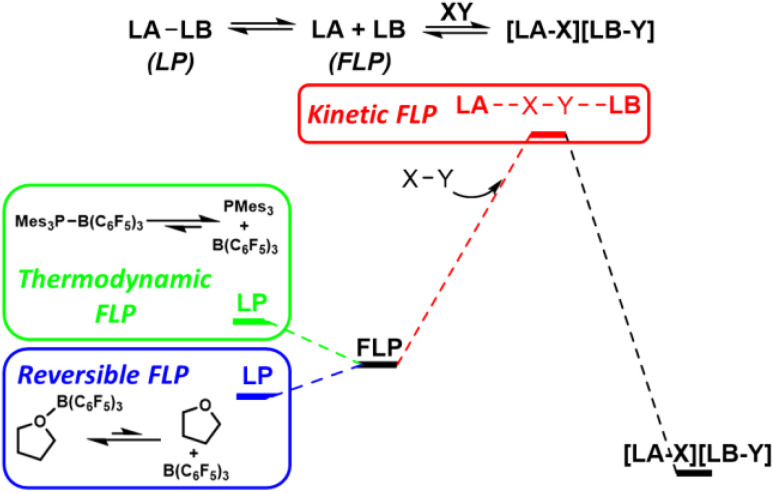
Thermodynamic FLP exhibit an FLP ground state. This provides a thermodynamic platform to enhance reactivity. Kinetic FLP cooperate synergistically to activate bonds through concerted transition states involving both the Lewis acid and Lewis base. LB = Lewis base, LA = Lewis acid, LP = Lewis pair.

The term ‘frustrated Lewis pair’ (FLP) was introduced in 2007, however, the FLP concept is under ongoing refinement.^15^ Early examples of main group Lewis acids and bases that failed to form stable observable Lewis adducts can be considered ‘thermodynamic FLP’ where the ground state of the Lewis pair is the frustrated form. However, much interest has arisen in the ability of FLPs to reduce activation barriers for bond cleavage through cooperative concerted transition states involving both Lewis acid components.^16^ Such systems can be considered ‘kinetic FLP’. It is important to note that an FLP system may be thermodynamic and/or kinetic, and that certain reaction advantages will arise from both of these aspects. Thermodynamically preferred Lewis pairs with a kinetically accessible FLP state have also exhibited kinetic FLP reactivity and have been termed ‘reversible FLPs’ (Fig. 7).

Seminal reports on FLP systems focused on main group element bases/acids due to their unheralded reactivity (*e.g.* the first examples of metal-free reversible dihydrogen cleavage). Recognized FLP systems have been expanded to include alkali metal, transition metal and even single atom acids and bases.^17^ Initial reactivity of FLP systems focused on the cleavage of a range of hydrogen element bonds (*e.g.* H–H, H–C, H–Si, H–B, H–O, H–N, H–Cl) but more recently activation of bonds in CO, N_2_ and CO_2_ (*inter alia*) has been demonstrated.^18^ Despite the apparent ability of FLP systems to mimic single site transition metal catalysts, very few reports exist for FLP activation of carbon halogen positions, despite C–X activation being a pillar of transition metal reactivity.^19^

## FLP mediated C–F activation

**Fig. 8:**
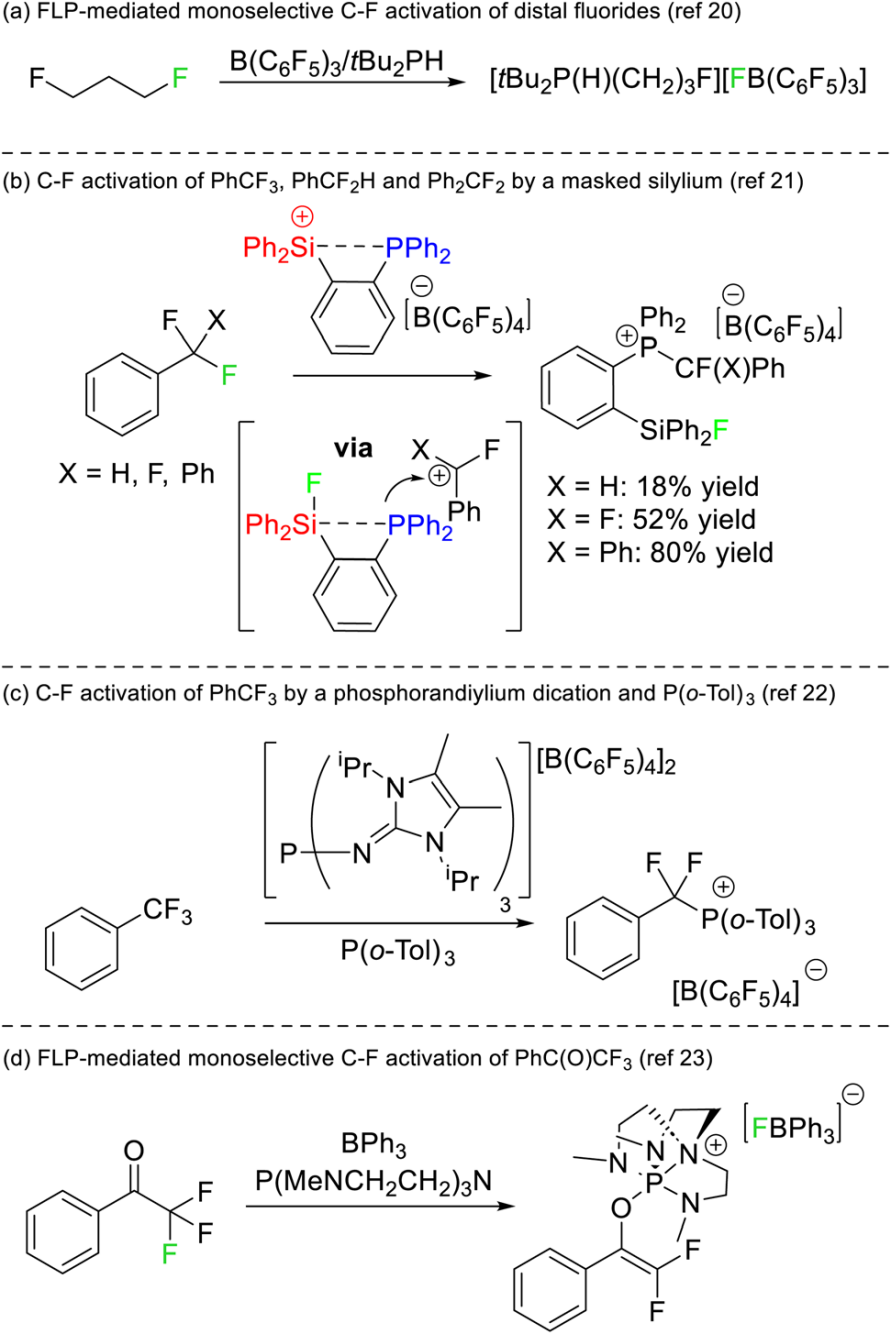
(a) The first report of controlled monoselective C–F bond activation in a polyfluoroalkane by an FLP. (b) Selective C–F bond activation by a phosphine masked silylium Lewis acid. (c) Selective activation of PhCF_3_ by a phosphorus(v) Lewis acid and P(*o*-Tol)_3_. (d) FLP activation of 2,2,2-trifluoroacetophenone by an FLP to generate a difluoroenolate product.

The first instance of C–F bond activation induced by an FLP was reported by Stephan in 2012.^20^ Activation of fluoromethyl groups with B(C_6_F_5_) and P^t^Bu_3_ resulted in phosphonium fluoroborate salts of the type [RP^t^Bu_3_][BF(C_6_F_5_)_3_]. Notably, the substrate 1,3-difluoroproporane was activated using B(C_6_F_5_)_3_ and PH^t^Bu_2_ to generate [F(CH_2_)_3_PH^t^Bu_2_][BF(C_6_F_5_)_3_] almost quantitatively where only a single C–F reacted with the FLP (Fig. 8a). Although the two fluorine atoms reside on different carbon positions, this report remains the first example of a monoselective activation of chemically equivalent positions in a polyfluoroalkane by an FLP.

Stephan later reported that a silylium phosphine adduct was capable of single C–F bond activation in trifluoromethylbenzene, difluoromethylbenzene and difluorodiphenylmethane (Fig. 8b).^21^ The silylium acted as both the Lewis acid and a thermodynamic sink for the liberated fluoride (in the formation of a silyl fluoride product), while the concomitantly generated carbocation was captured by the phosphine motif to generate a fluoroalkylphosphonium product. DFT studies revealed that dissociation of the phosphine was not required for the silicon Lewis acid to abstract fluoride, and as such the system is not technically a thermodynamic nor a kinetic FLP. Stephan also demonstrated that the activated fluorocarbons could be released from the phosphine Lewis base in the presence of hydroxide representing a formal monoselective hydrodefluorination reaction. A similar selective C–F bond activation of PhCF_3_ utilizing a phosphorus(v) dication as a strong Lewis acid and P(*o*-Tol)_3_ as a Lewis base was reported by Dielmann in 2019 (Fig. 8c).^22^

Stephan also reported on the FLP mediated monoselective activation of 2,2,2-trifluoroacetophenone (Fig. 8d).^23^ It was known that the electron rich phosphine P(NMe_2_)_3_ reacted with trifluoroacetophenone to give a mixture of products.^24^ Stephan utilized the electronically similar but structurally constrained phosphine P(MeNCH_2_CH_2_)_3_N in combination with BPh_3_ to both stabilize the phosphonium (which resulted from reduction of the carbonyl position) and to sequester fluoride liberated in the reaction. As such, the conversion of trifluoroacetophenone to a difluoroenolate in a high yield of 87% was possible.

**Fig. 9:**
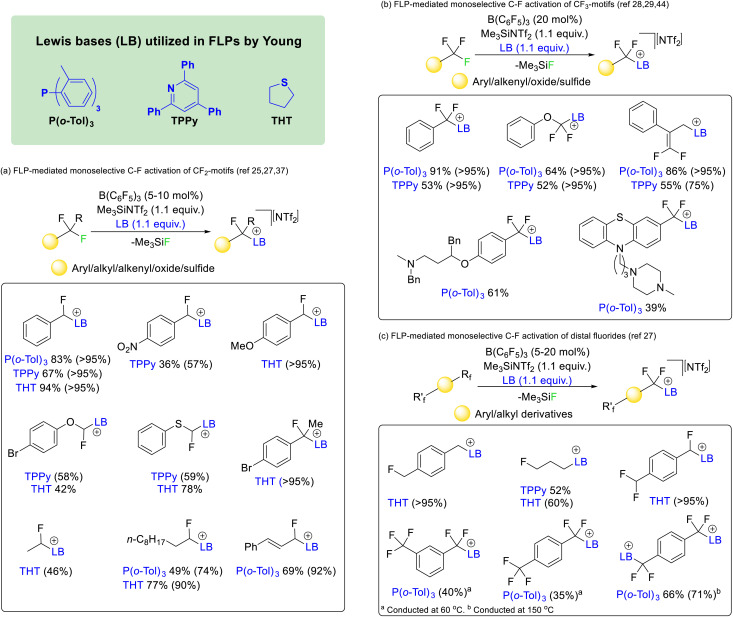
FLP mediated activation reported by Young utilizing phosphine, pyridine and sulfide Lewis bases. Reactions that are catalytic in Lewis acid are possible with the use of a fluoride sequestering agent (*e.g.* Me_3_SiNTf_2_). The reaction works for difluoromethyl, trifluoromethyl and distal difluoride groups in a variety of chemical environments.

In 2018 Young utilized borane and phosphine FLPs to activate difluoromethyl positions in a range of fluorocarbons (Fig. 9a).^25^ The reaction was found to work with affordable and commercially available Lewis acid/base mixtures such as boron trifluoride and triphenylphosphine. However, the most effective phosphine proved to be P(*o*-Tol)_3_ in combination with B(C_6_F_5_)_3_. The reaction was later made catalytic in Lewis acid with the addition of Me_3_SiNTf_2_ as a fluoride sequestering agent^26^ and extended to the nitrogen Lewis base 2,4,6-triphenylpyridine (TPPy) and the sulfide bases tetrahydrothiophene (THT) and dimethylsulfide.^27^ It was found that the reaction was capable of selectively activating C–F bonds of difluoromethyl groups hosted by a range of chemical supports including aryl, heteroaryl, alkyl, alkenyl, oxide and sulfide groups.

Similar reaction conditions allowed the selective activation of trifluoromethyl groups, although the reaction was found to only proceed with phosphine and pyridine Lewis bases (*i.e.* P(*o*-Tol)_3_ and TPPy) rather than sulfide bases (Fig. 9b).^28^ The reaction was found to be compatible with aryl, heteroaryl, alkenyl, oxide and sulfide supported trifluoromethyl groups. However, in contrast to the activation of difluoromethylalkenyls (that gave geminal substitution), activation of α,α,α-trifluoromethylstyrenes resulted in S_N_2′ substitution and generation of a difluoroolefin product.^29^

The concept was also extended to chemically equivalent distal fluorides. As such selective activation of a single C–F bond in alkyl and aryl linked monofluoromethyl, difluoromethyl and trifluoromethyl groups was possible (Fig. 9c).^27^

## Mechanistic studies on FLP mediated C–F bond activation

A number of mechanistic studies have been conducted to reveal the active reaction pathways for FLP mediated C–F bond activation. Fernandez conducted theoretical studies on the FLP system reported by Young.^30^ He found that an FLP mechanism was preferred over an S_N_1 type mechanism and identified a 5-coordinate carbon-centred structure as a key intermediate. Chatteraj later performed theoretical studies on a simplified lutidine/alane system (that had not been experimentally authenticated) with a similar calculated FLP pathway to that of Fernandez.^31^

**Fig. 10:**
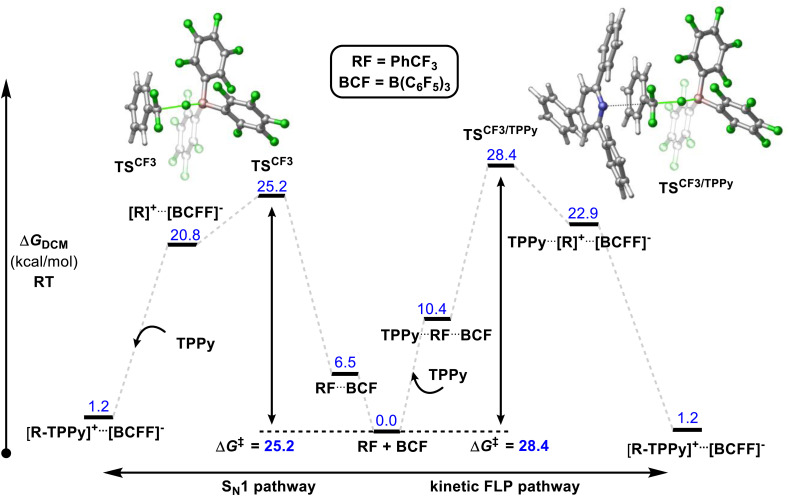
Young reported that a Lewis acid assisted S_N_1 mechanism was found to be experimentally and theoretically more plausible than a kinetic FLP pathway. However, a thermodynamic FLP ground state was also found to be critical for reactivity, with the reversible FLP of THT/B(C_6_F_5_)_3_ unable to activate benzotrifluorides while activation of benzotrifluorides occurred under ambient conditions with the thermodynamic FLPs TPPy/B(C_6_F_5_)_3_ and P(*o*-Tol)_3_/B(C_6_F_5_)_3_. Level of theory: PCM(DCM)-B3LYP-D3/Def2TZVPP//PCM(DCM)-DB3LYP-D3/Def2SVP (quasi-harmonic entropic correction). See ref. 32 for details.

In contrast, Young reported a combined experimental and theoretical study that corroborated an S_N_1 pathway.^32^ Young's study found that the reaction of benzotrifluorides and benzodifluorides with a variety of FLP systems was independent of Lewis base concentration, and a Hammett plot analysis revealed large negative *ρ*-values (−3 to −7) consistent with an S_N_1 process for the C–F bond activation step. The proposed theoretical model supported this mechanism with a kinetic barrier of 25.2 kcal mol^−1^ for the activation of PhCF_3_ with B(C_6_F_5_)_3_ and TPPy *via* an S_N_1 pathway *versus* a barrier of 28.4 kcal mol^−1^ for an FLP pathway (Fig. 10). Despite the preference for an S_N_1 pathway over a kinetic FLP pathway, Young determined that a thermodynamic FLP was necessary for the reaction to proceed under practical conditions with Lewis pair formation between TPPy and B(C_6_F_5_)_3_ being endergonic by 3.0 kcal mol^−1^. Indeed, it was found that THT and B(C_6_F_5_)_3_ formed a reversible FLP with a 1–2 kcal mol^−1^ thermodynamic penalty for Lewis pair dissociation that inhibited reactivity with benzotrifluorides.

Young's theoretical model also revealed that the carbocation intermediate accessed *via* an S_N_1 pathway is relatively unstable with respect to the kinetic barrier for C–F bond cleavage meaning that the barrier to nucleophilic attack of this intermediate rivalled that of C–F bond activation as the rate limiting step. This result likely explains why efforts by others to activate benzotrifluoride using B(C_6_F_5_)_3_ in combination with poorer nucleophiles has failed. For example, B(C_6_F_5_)_3_ failed to catalyse the hydrodefluorination and Friedel–Crafts arylation of benzotrifluoride.^33^

**Fig. 11:**
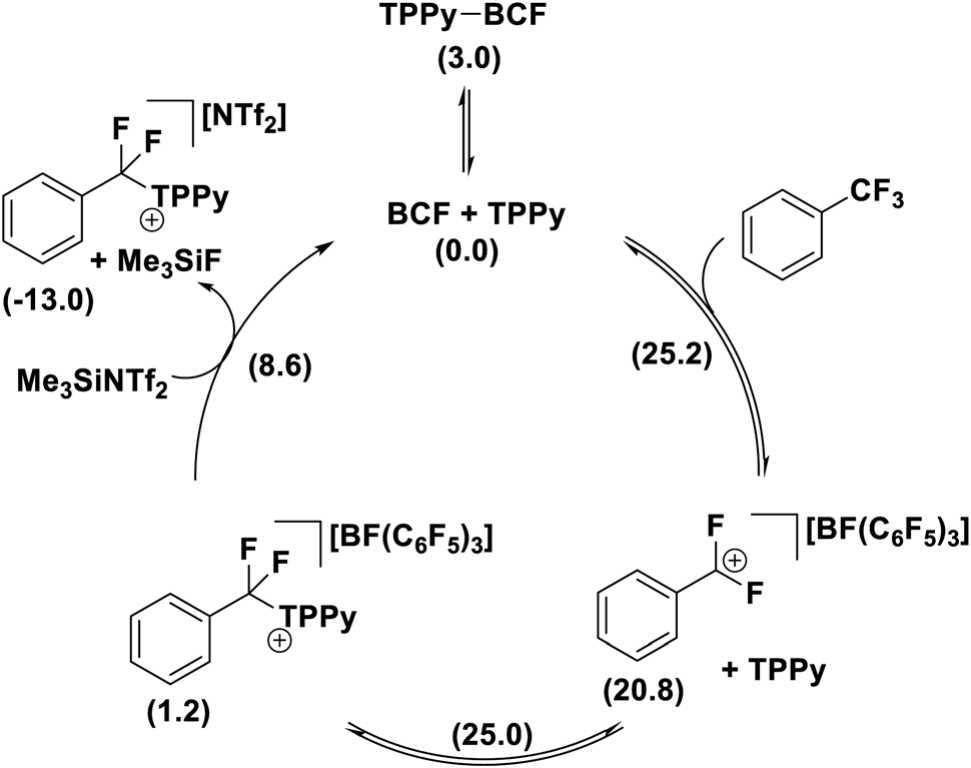
The products of FLP mediated selective C–F bond activation are in equilibrium with the starting materials and require a fluoride sequestration agent to facilitate catalysis. Free energies in kcal mol^−1^ given in parentheses.

The formation of the product [PhCF_2_(TPPy)][BF(C_6_F_5_)_3_] was found to be slightly endergonic by 1.2 kcal (*versus* the FLP ground state) and the low kinetic barrier allows for a dynamic equilibrium. For substrates where the equilibrium lies towards the starting materials, the addition of a fluoride sequestering reagent (*e.g.* Me_3_SiNTf_2_) is requisite for reaction turn-over and productive reactivity (Fig. 11). Importantly, Young examined subsequent defluorination steps and discovered that the kinetic barrier for defluorination of the cationic fluorocarbon salt fragments was substantially raised (even for distal C–F positions). For example, the second defluorination event for PhCF_2_H using B(C_6_F_5_)_3_ and P(*o*-Tol)_3_ was 6 kcal mol^−1^ higher in energy than the first defluorination step and 13.6 kcal mol^−1^ higher in energy than defluorination of BnF.^32^ An increased kinetic barrier for defluorination of a cationic fluorocarbon further supports an S_N_1 mechanism.

Similar to Young's proposed Lewis acid assisted S_N_1 mechanism, Stephan conducted DFT studies on his silylium mediated C–F bond activation (Fig. 8b) that suggested a Lewis acid assisted S_N_1 mechanism.^21^ In contrast to Young's system, the ground state of Stephan's system was a strained 4-member silyl phosphonium ring. The ability of silicon to accommodate higher coordination (*cf.* boron) resulted in fluoride abstraction by silicon prior to phosphine decoordination, and as such there was no thermodynamic penalty required for silicon–phosphorous dissociation (*i.e.* a reversible FLP wasn't necessary for reactivity). The resultant intermediate carbocation generated after C–F bond activation was subsequently captured by the liberated phosphine.

## Applications

FLP mediated monoselective C–F bond activation allows the capture of the activated fluorocarbon fragment with a range of Lewis base partners. As stated above, these Lewis base partners can play a pivotal role in the activation reaction (through the formation of thermodynamic FLPs), however, such Lewis bases also act as nucleofuges for further reactivity. Indeed, deconvoluting the C–F bond functionalization process into ‘activation’ and ‘functionalization’ steps allows for an extremely extensive array of functionalization possibilities. Further, the relative stabilities of the salts resulting from C–F bond activation allows for ‘customization’ of the activation reaction based on the reactivity of the cationic fluorocarbon fragment and the desired functionalization. With respect to heterolysis, phosphonium salts are more stable than pyridinium salts and pyridinium salts are more stable than sulfonium salts, while a higher resonance stability of the cationic fluorocarbon fragment leads to a less stable salt.

**Fig. 12:**
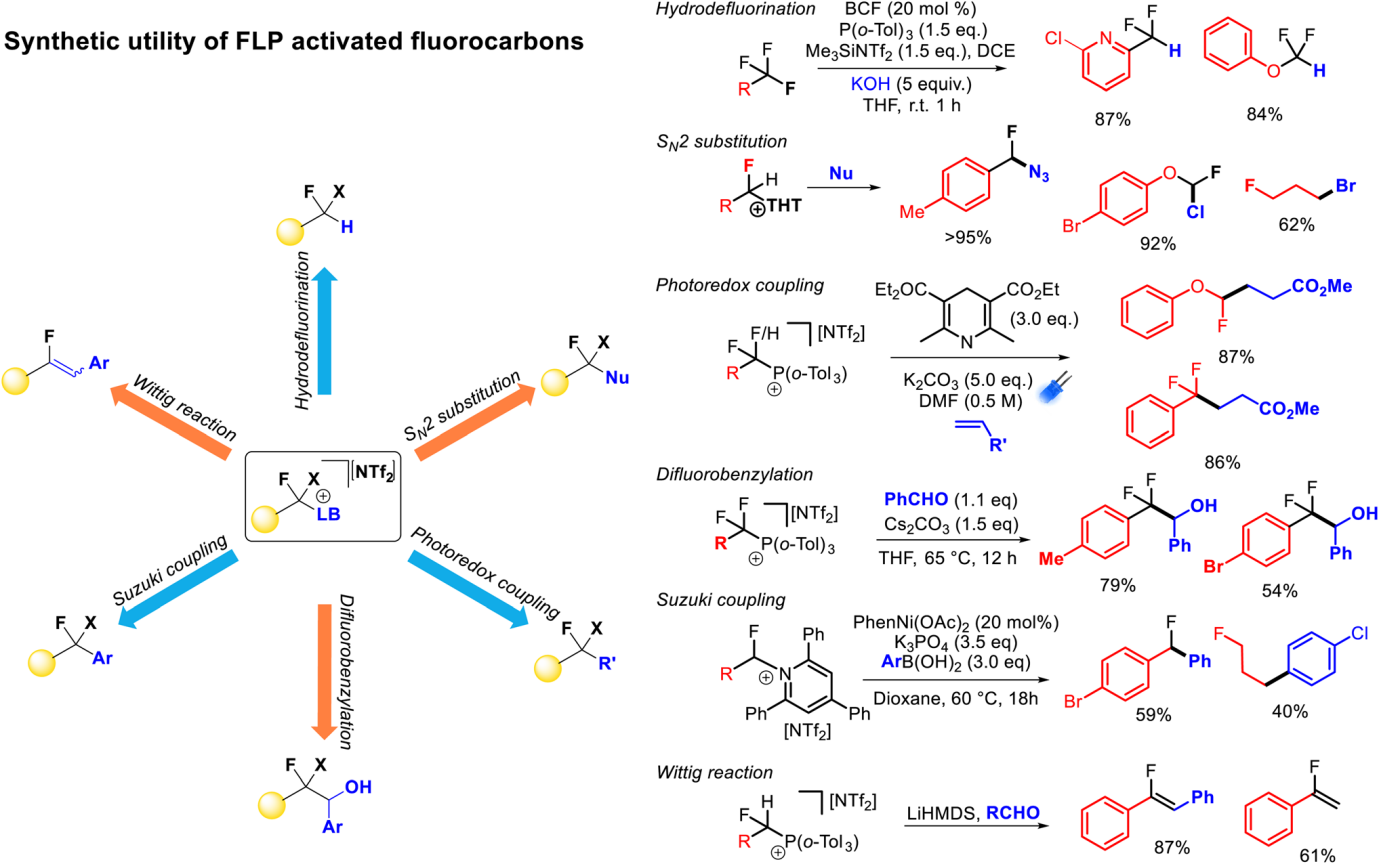
A large number of functionalization reactions are possible post C–F bond activation. Judicious choice of Lewis base allows for specific functionalization. Thus far, formal hydrodefluorination, nucleophilic substitution, photoredox alkylation, nucleophilic transfer, Suzuki–Miyaura coupling and Wittig olefination have been demonstrated as post-activation functionalisations.

As described above, FLP systems provide a general method of selective C–F bond activation for a wide selection of polyfluoroalkanes. Indeed, apart from spanning multiple substrate classes that are specific to other activation approaches (*e.g.* difluoromethyl(hetero)arenes, trifluoromethyl(hetero)arenes, trifluoromethylalkenes, trifluoromethylketones), FLP mediated selective C–F bond activation allows derivatization of unique substrates that are resistant to activation by any other method (*e.g.* difluoroalkanes, difluoro(thio)methyoxides, trifluoro(thio)methyoxides). The ability to install sulfides, phosphines and pyridines as nucleofuges provides the ability for a vast array of functionalization opportunities allowing convenient access to a diverse range of derivatives from a common fluorocarbon starting material (Fig. 12).

The derivatization of phosphonium, sulfonium and pyridinium salts is highly developed for non-fluorinated reagents, and (in-principle) such chemistry is applicable to the products of FLP monoselective C–F bond activation. Notably, Katritzky salts (containing TPPy) have recently become popular in the redox coupling community to install alkyl, alkenyl, aryl and boryl groups (*inter alia*),^33^ alkyl sulfonium salts were shown to be excellent electrophilic partners in palladium catalysed coupling chemistry by Libeskind^34^ and alkyl phosphonium salts have a rich coupling chemistry history in Wittig olefination and redox alkylation.^35,36^

Stephan demonstrated formal hydrodefluorination of PhCF_3_, PhCF_2_H and Ph_2_CF_2_*via* phosphonium salts.^21^ However, Young has expanded on this and exemplified the synthetic utility of the FLP approach to selective C–F bond activation.^25–29^ Young has demonstrated that the installation of phosphonium and pyridinium groups allows for the fluorocarbon fragment to act as a radical or anionic nucleophile in alkylation and benzylation reactions in a similar fashion to reductive strategies. However, given the lower energies required for C–N and C–P bond cleavage (as compared to C–F bond cleavage) a greater functional group tolerance is possible using FLP activated salts as compared to benzotrifluorides activated by reductive approaches directly. For example, aryl bromides have been shown to be incompatible with alkyl redox coupling conditions,^12^ while Young has demonstrated high yields of redox alkylation products from phosphonium fluorobenzyl salts featuring bromo groups.

Young has also shown that activated fluorocarbon fragments can be utilized as electrophilic partners (Fig. 12). As such, a general approach to monoselective nucleophilic substitution of fluoride in polyfluoroalkanes has been realized.^27,28^ Young has demonstrated that both sulfide and pyridine groups are readily displaced by a range of nucleophiles including halides, azides, cyanide, thiocyante, nitrate, oxides, sulfides, carboxylates, N-heterocycles, pyridines, phosphines and amines (*inter alia*). Young has shown that the basicity of the nucleofuge (*e.g.* THT, TPPy) can be matched to both the desired nucleophile and the fluorocarbon substrate to provide optimum reaction yields.

Young has also demonstrated that Katritzky salts can be utilized in metal catalysed couplings.^27^ For example, nickel and palladium were shown to catalyse Suzuki–Miyori couplings with arylboronates to provide access to fluorinated diaryl methanes. In principle, Negishi couplings, reductive couplings and borylations (*inter alia*) are accessible using a similar approach.^34*b*^

Lastly, fluorinated alkyl phosphonium salts have been shown to facilitate Wittig olefination reactions.^25^ Due to the requirement of an *α*-hydrogen preceding ylide formation, this type of functionalization is restricted to difluoromethyl or fluoromethyl substrates. The presence of an *α*-fluoro group facilitates ylide formation with moderate strength bases (*e.g.* lithium amides). Generally, fluoroalkenes are challenging to access, and this protocol allows for one-pot synthesis of fluoroolefines directly from difluoromethylalkanes in good yield and selectivity.

## Stereoselective fluoroalkane synthesis

As discussed above, displacement of pyridine and sulfide Lewis bases from activated fluorocarbon fragments is a facile process. As such, exchange of Lewis bases readily occurs in solution *via* an S_N_1 process. Recently, Young has reported that this process allows stereochemical control in the activation of enantiotopic difluorides.^37^ The majority of synthetic approaches to access stereoenriched fluorocarbon centres rely upon ‘bottom-up’ approaches that need to introduce fluorine, whereas stereoselective FLP mediated C–F bond activation is a ‘top-down’ approach that selectively removes fluorine to generate a stereoenriched fluorocarbon centre.^38^ Apart from the attraction of using pre-existing polyfluorocarbons, this method also allows the generation of fluorocarbon centres that are not accessible using other developed approaches (*e.g.* centres that cannot be generated from stereoselective electrophilic fluorination reagents, fluoride addition or elaboration).

**Fig. 13:**
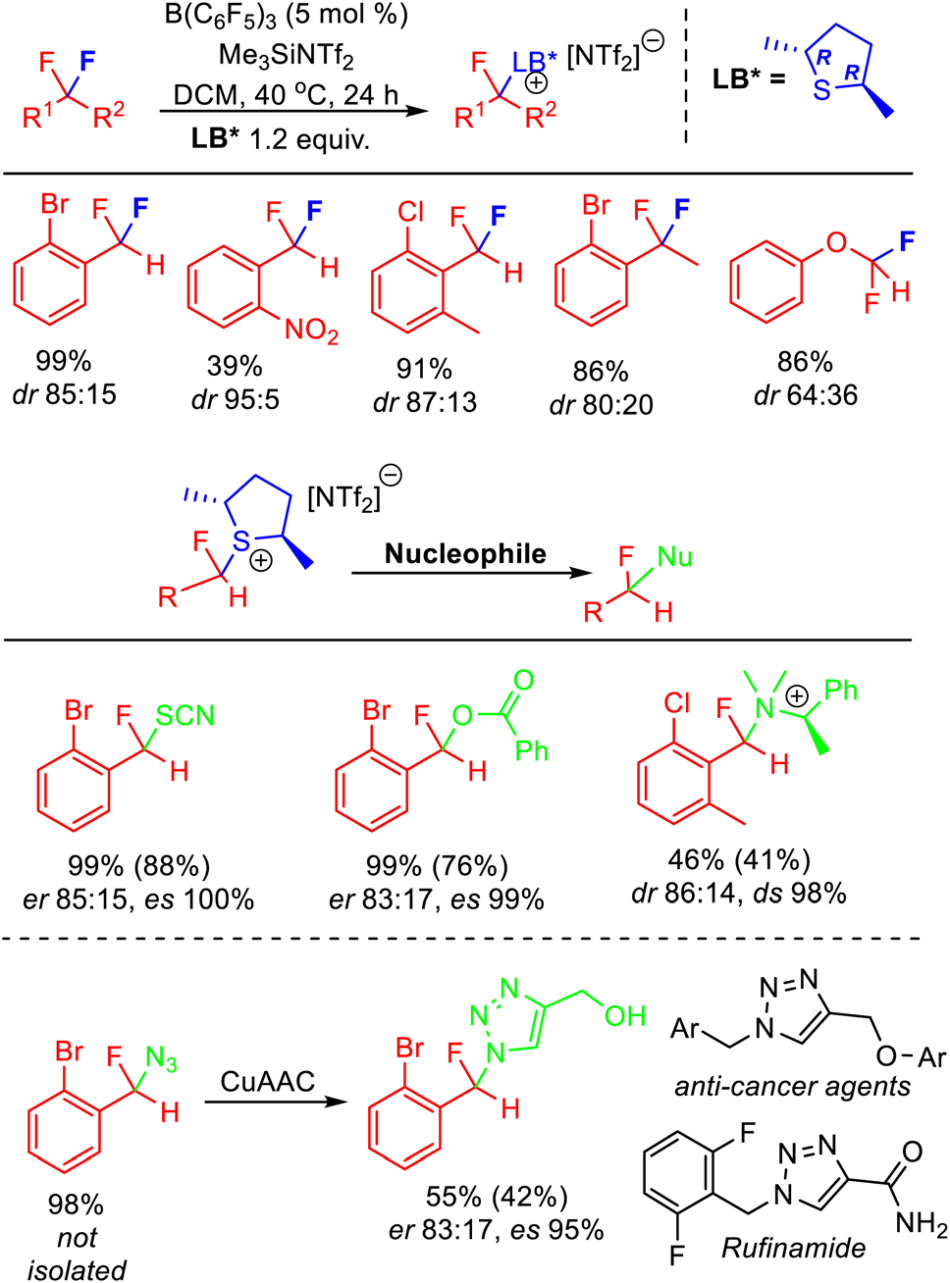
Stereoselective FLP C–F bond activation enabled through the use of a chiral Lewis base partner. The use of an enantiopure chiral base allows the generation of enantiomerically enriched products through S_N_2 substitution of the diastereomeric activation salts. Yields based on NMR, isolated yields in parentheses. See ref. 37 for details.

The stereoselective FLP mediated C–F bond activation reactions reported by Young relied upon the use of chiral Lewis bases, giving rise to diastereomeric activation products (Fig. 13). As such, the rate of chiral Lewis base exchange and the free energy difference between the diastereomers controlled the selectivity of the reaction. It was found that difluoromethylarenes containing ortho substituents combined with chiral dialkylsulfides gave optimum results, with selectivity as high as *dr* = 95 : 5 observed. Utilizing enantiopure (*R*,*R*)-2,5-dimethylthiolane gave rise to epimers that could be derivatized *via* S_N_2 substitution reactions to generate enantioenriched fluorocarbons. Young demonstrated how this method could be utilized to access a fluorinated analogue to Rufinamide in *ca* 70% ee. Enantioenriched benzylfluorides subtended by heteroatoms are difficult to generate using existing ‘bottom-up’ enantioselective fluorination approaches.

## Direct access to radiolabelled fluorocarbons

Fluorine finds a unique role in radiology where the half-life and emission energy of the synthetic isotope fluorine-18 render it the most practical for positron emission tomography (PET) imaging.^39^ Apart from well-established applications in diagnostic medicine, PET imaging has been shown to be a powerful tool in pharmacokinetics and drug development.^40^ Difluoromethyl and trifluoromethyl groups have become a pillar of modern pharmaceuticals,^41^ thus the ability to generate fluorine-18 isotopologues of drugs under development would accelerate their metabolic studies.

‘Bottom-up’ approaches to incorporating fluorine-18 into CF_2_H and CF_3_ positions suffer from the need to implement custom synthetic pathways to install fluorine-18 at a late stage. This contrasts with synthetic strategies to non-labelled compounds that install CF_3_ and CF_2_H groups early in the synthetic route.^42^ As stated above, FLP mediated removal of fluoride from difluoromethyl and trifluoromethyl positions is a dynamic equilibrium, where fluoride (in the form of [BF(C_6_F_5_)_3_]^−^) displaces the Lewis base on the activated fluorocarbon fragment to regenerate the fluorocarbon starting material. As such, [^18^F]F^−^ can be utilized as a fluoride source to generate isotopologues from the non-labelled target in a two-step process. Such a synthetic strategy not only allows the installation of CF_3_ and CF_2_H units early in the synthetic route but allows direct use of the target compound as a starting material, greatly simplifying the radiosynthesis of fluorine-18 labelled CF_3_ and CF_2_H groups in a wide range of chemical settings.^43^

**Fig. 14:**
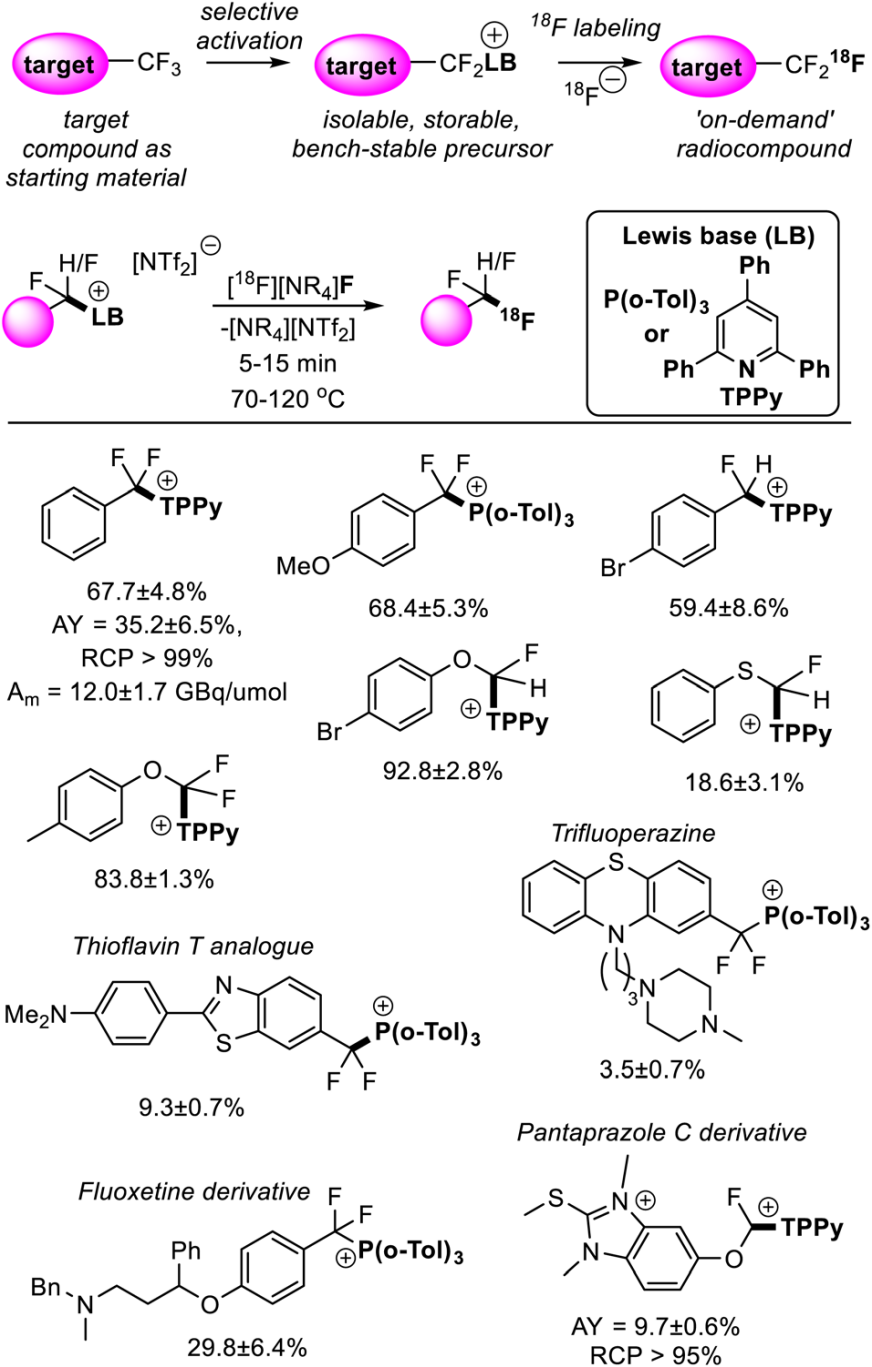
Synthesis of fluorine-18 labelled CF_3_ and CF_2_H groups is possible *via* FLP selective activation followed by Lewis base substitution with [^18^F]F^−^. This methodology greatly simplifies the radiosynthesis of the fluorine-18 isotopologues as it allows the non-labelled target compound to be used as a starting material. Yields correspond to radiochemical conversions (RCC). See ref. 44 for details.

Young reported on the FLP mediated C–F bond activation and isolation of a range of difluoromethyl and trifluoromethyl containing compounds including bioactive targets (and commercially available pharmaceuticals). These were then utilized in radiofluorination to generate the radiolabelled targets (Fig. 14).^44^ The radiofluorination step was shown to proceed quickly under mild conditions (5–15 minutes, 70–120 °C) and demonstrated good functional group tolerance. Given the mild conditions of the radiofluorination step, good radiochemical yields and molar activities were achieved. For example, a sample of [^18^F]PhCF_3_ was isolated in a non-decay corrected activity yield (AY) of 35.2 ± 6.5%, a non-decay corrected molar activity (*A*_m_) of 12.0 ± 1.7 GBq μmol^−1^ and a radiochemical purity (RCP) greater than 99% starting from low initial activities (3–5 GBq). Other approaches to generate fluorine-18 labelled CF_3_ groups that require harsher reaction conditions generally suffer from fluoride scrambling and *A*_m_ greater than 10 GBq μmol^−1^ are difficult to achieve starting from low initial activities.

## Conclusion

Fluorocarbons have proven invaluable chemicals that are required for a range of modern technologies, and their use will continue despite any concerns over their environmental persistence. Consequently, the need to access a diverse variety of second-generation fluorocarbons *via* selective C–F bond functionalization is well-recognised. FLP systems offer a unique solution to the problem of ‘over-defluorination’ in polyfluoroalkanes and allow selective activation of C–F bonds in CF_3_ and CF_2_R motifs supported by a wide range chemical supports including aryl, heteroaryl, alkyl, alkenyl, silyl, carbonyl, oxide and sulfide groups (*inter alia*). Notably, FLP systems have been shown to activate small fluorocarbon refrigerants (fluoroalkanes) selectively, a transformation not possible using other selective C–F bond functionalization approaches.

Combined experimental and theoretical studies by Stephan, Young, Fernandez and Chatterjee suggest that thermodynamic FLP systems are important platforms to promote reactivity but that fluorocarbon activation proceeds *via* a Lewis acid assisted S_N_1 mechanistic pathway. Further, these theoretical studies have quantified the elevation of the kinetic barrier for over defluorination steps, uncovering the basis for the highly monoselective reaction.

Importantly, products of FLP mediated C–F bond activation can be functionalized with pre-established coupling chemistry protocols that can (in-principle) install almost any functional group. Hydrogenolysis, alkylation, arylation, olefination, electrophilic transfer and nucleophilic transfer functionalizations have all been demonstrated.

Thus far, only a small sample of FLP systems have been explored in C–F bond activation. These include archetypal Lewis acids B(C_6_F_5_)_3_, Al(C_6_F_5_)_3_ and BF_3_, as well as newly developed phosphorus(v) dicationic and silylphosphonium Lewis acids. Lewis base exploration has been a little more adventurous, with pyridines, phosphines and thioethers all utilized in FLP mediated C–F bond activation. The importance of FLP combinations for both the activation steps and subsequent functionalization has become apparent, and FLP components can be customized based on the characteristics of the C–F bond to be targeted.

Utilizing chiral Lewis bases, FLP mediated C–F bond activation also allows stereoselective desymmetrization of enantiotopic difluorides. This provides a rare example of a ‘top-down’ approach to stereoenriched fluorocarbon centres. Stereoselective FLP mediated C–F bond activation provides a complementary synthetic strategy to existing stereoselective fluorination methods and provides access to stereoenriched fluorocarbon centres that would otherwise be difficult to generate.

The products of FLP mediated C–F bond activation have also been demonstrated to allow direct access to fluorine-18 labelled CF_3_ and CF_2_H groups. This allows the use of target compound as the starting material and can greatly simplify radiosynthesis of pharmaceutical isotopologues utilized in pharmacokinetic studies.

A multitude of opportunities present themselves for future development of FLP mediated C–F bond activation. Proof-of-principle for the use of FLP mediated C–F bond activation in radiochemistry, stereoselective synthesis and C–F derivatization have been reported (and discussed above) but the development and application of these chemistries is on-going. Further, the ability of FLPs to mimic transition metal chemistry may allow FLPs to act as multifunctional catalysts in cascade reactions. For example, the ability of FLPs to (selectively) activate both C–F and H–H bonds may allow for hydrodefluorination reactions that utlilise hydrogen gas as opposed to molecular hydrides. Given that the larger area of FLP chemistry has been well-developed over the last two decades, activation of fluorocarbons could be coupled with heterogeneous FLPs,^45^ frustrated radical pairs (FRPs),^46^ transition metal FLPs^17–19^ and the FLP activation of small molecules (*e.g.* N_2_, CO, CO_2_)^16^ to enhance the utility of FLP mediated C–F bond activation. Given that FLP catalysed activation of benzotrifluoride was only demonstrated in 2020, the ability of FLP systems to efficiently and conveniently generate second generation fluorocarbons is only beginning to be realized by the greater chemical community and the future contributions that FLPs will make to selective C–F bond activation look set to explode.

## Data availability

All data to support the assertions in this perspective can be found in the relevant references.

## Author contributions

K. L. and R. D. Y. jointly compiled the relevant literature and co-wrote the manuscript.

## Conflicts of interest

There are no conflicts to declare.

## Supplementary Material
